# Bioactivities and Mechanisms of Action of Diphyllin and Its Derivatives: A Comprehensive Systematic Review

**DOI:** 10.3390/molecules28237874

**Published:** 2023-11-30

**Authors:** Wen Hou, Le-Jun Huang, Hao Huang, Sheng-Lan Liu, Wei Dai, Zeng-Min Li, Zhen-Yu Zhang, Su-Ya Xin, Jin-Yang Wang, Zi-Yun Zhang, Xi Ouyang, Jin-Xia Lan

**Affiliations:** 1 College of Pharmacy, Gannan Medical University, Ganzhou 341000, China; twenhou@163.com (W.H.); hhuang@gmu.edu.cn (H.H.); liushl5@gmu.edu.cn (S.-L.L.); daiwei@gmu.edu.cn (W.D.); zzy3110734209@163.com (Z.-Y.Z.); 17870302921@163.com (S.-Y.X.); jin17867983095@163.com (J.-Y.W.); 19324828384@163.com (Z.-Y.Z.); 15350320244@163.com (X.O.); 2 College of Public Health and Health Management, Gannan Medical University, Ganzhou 341000, China; 3 College of Rehabilitation, Gannan Medical University, Ganzhou 341000, China; 18379811337@163.com; 4 Laboratory Animal Engineering Research Center of Ganzhou, Gannan Medical University, Ganzhou 341000, China; lizengmin@gmu.edu.cn

**Keywords:** review, natural product, diphyllin, V-ATPase, anti-tumor, anti-virus

## Abstract

Natural products are treasure houses for modern drug discovery. Diphyllin is a natural arylnaphthalene lignan lactone isolated from the leaf of *Astilboides tabularis*. Studies have found that it possesses plenty of bioactivity characteristics. In this paper, we reviewed the structure, bioactivity, and mechanism of action of diphyllin and its derivatives. The references were obtained from PubMed, Web of Science, and Science Direct databases up to August 2023. Papers without a bio-evaluation were excluded. Diphyllin and its derivatives have demonstrated V-ATPase inhibition, anti-tumor, anti-virus, anti-biofilm, anti-inflammatory, and anti-oxidant activities. The most studied activities of diphyllin and its derivatives are V-ATPase inhibition, anti-tumor activities, and anti-virus activities. Furthermore, V-ATPase inhibition activity is the mechanism of many bioactivities, including anti-tumor, anti-virus, and anti-inflammatory activities. We also found that the galactosylated modification of diphyllin is a common phenomenon in plants, and therefore, galactosylated modification is applied by researchers in the laboratory to obtain more excellent diphyllin derivatives. This review will provide useful information for the development of diphyllin-based anti-tumor and anti-virus compounds.

## 1. Introduction

**Figure 1:**
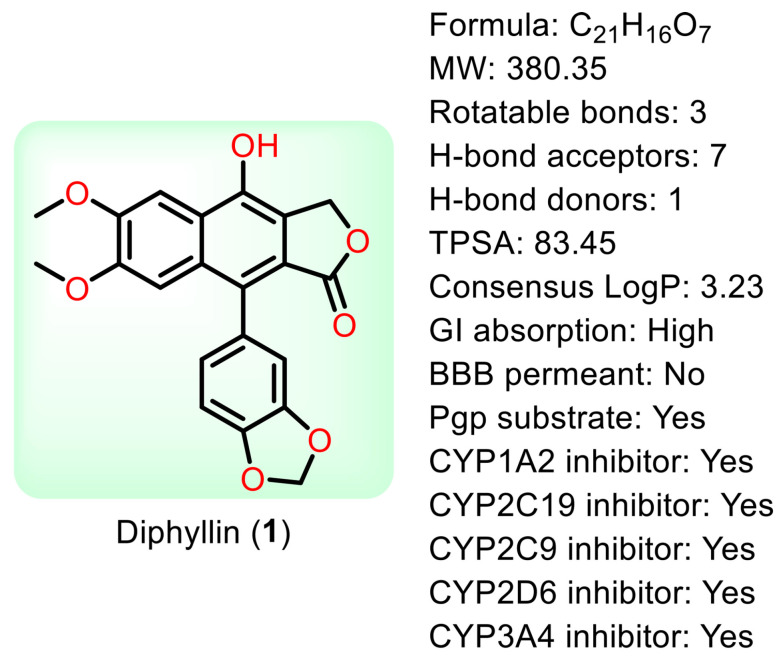
The structure of diphyllin and some parameters obtained from SwissADME.

**Figure 2:**
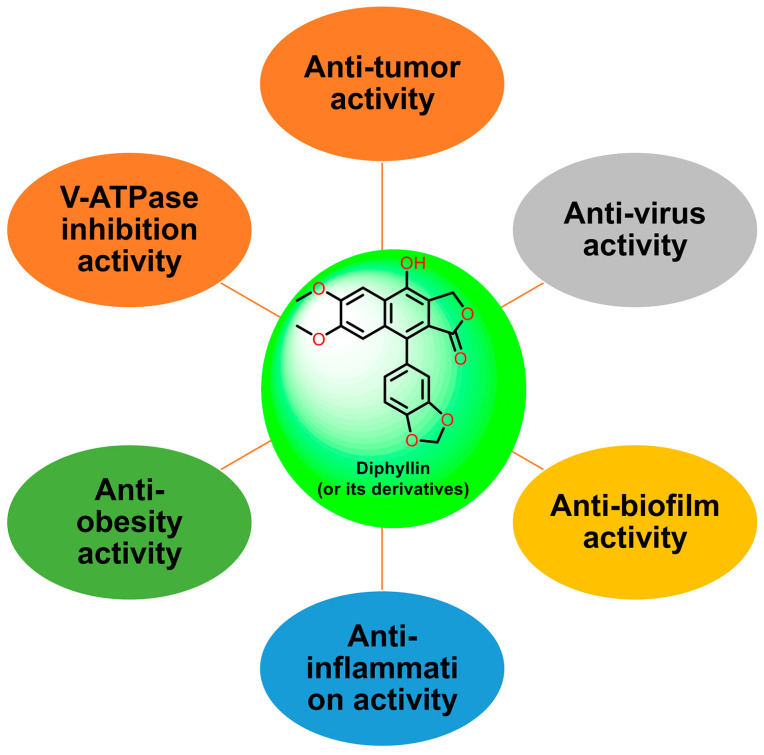
The bioactivities of diphyllin or its derivatives.

Natural products are renewable sources for drug discovery. The structural diversity of natural products and their easy combination with biological macromolecules determine their participation in life control. They have incomparable advantages in the life regulation process, and they also occupy an irreplaceable position in the research and development of new drugs. From the discovery of traditional Chinese medicine to modern antibiotics, natural products have played a significant role. In modern drug development, natural products and their derivatives are always important sources of candidate drugs and lead compounds. According to statistics, nearly two-thirds of all drugs on the market from 1981 to 2019 were related to natural products [1]. An abundance of oxygen atoms in their complex structures endows them with advanced binding properties with drug targets [2]. Diphyllin (1) is an arylnaphthalene lignan lactone that can be isolated from many traditional medicinal plants [3,4]. There are seven oxygens in its structure (Figure 1), the molecular weight of which is 380.35. We conducted ADME predictions using free online services [5] (http://www.swissadme.ch/index.php, accessed on 1 November 2023). The results found that it had a high topological polar surface area (TPSA) value of 83.45, which indicated strong membrane permeability. The cLogP value (3.23) was also moderate, following the Lipinski’s Rule of Five. It was predicted to have high gastrointestinal absorption. However, the blood–brain barrier (BBB) permeant was low, implying that it can only arrive at the brain tissue with difficulty. Disappointingly, it was also predicted with liver microsomal enzymes inhibitory activity, which means that drug interactions with other enzymes occur easily. It is characterized by muti-bioactivities such as anti-virus, anti-tumor, anti-inflammatory, and antioxidant activities. However, some drawbacks, such as its relatively weak potency, low aqueous solubility, and poor metabolic stability, restrict its developmental prospects. Therefore, numerous studies have been conducted across the world in the pursuit of more appropriate active derivatives. The bioactivities of diphyllin (**1**) or its derivatives are outlined in Figure 2 and are detailed point by point below.

## 2. Anti-Tumor Activity

**Figure 3:**
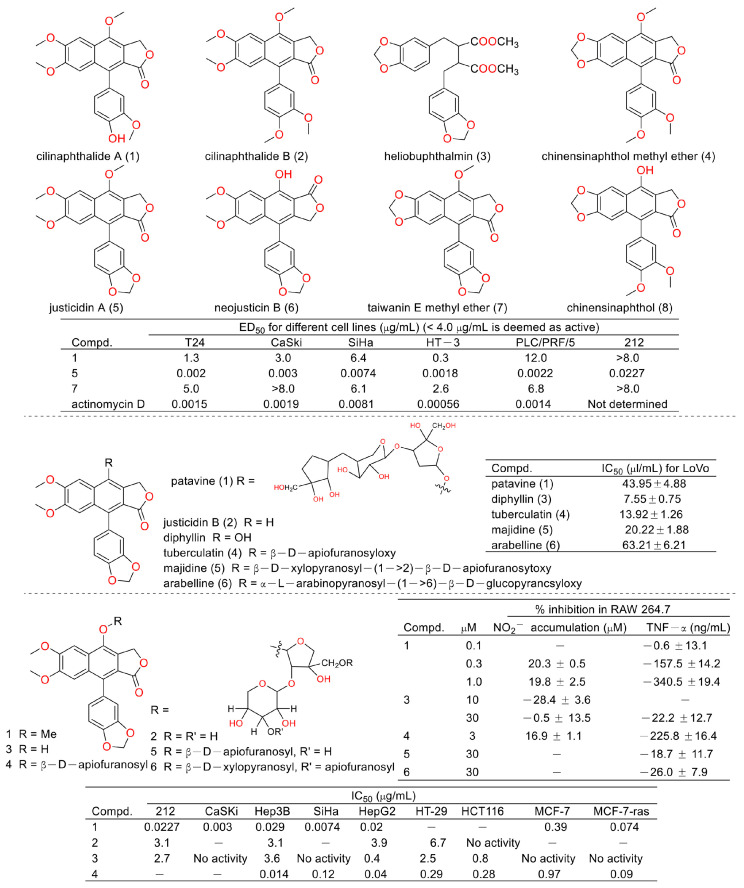
Structures and activities of diphyllin as well as its derivatives.

Cancer is the second leading cause of human death around the world [6,7]. Therefore, discovering anticancer agents to reverse this situation is imperative [8]. Early in 1999, Shiowhwa Day et al. published a paper in the Journal of Natural Products. They stated that the diphyllin analogues 1, 5, and 7 were cytotoxic against several cancer cell lines (e.g., T24 (bladder cancer cells), CaSKi (cervical cancer intestinal metastatic cells), SiHa (cervical squamous cells), HT-3 (cervical cancer cells), PLC/PRF/5 (liver cancer Alexander cells), and 212 (brain neuroblastoma cells)) (Figure 3), among which compound **5** was prominent (IC_50_ values were several pico per milliliter). The diphyllin analogues 2, 4, 6, and 8 were not active for cancer cell lines [9]. In 2002, Gabbriella Innocenti et al. isolated six compounds from shoot cultures of Haplophyllum patavinum, and they found that diphyllin (compound **3**) displayed the best anti-human colon carcinoma (LoVo cell line) activity with an IC_50_ value of 7.55 μL/mL [10]. In the same year, Shiow-Hwa Day and co-workers delineated a novel 4-*O*-α-L-arabinopyranosyl-(1‴→2″)-β-D-apiofuranosyldiphyllin (2), also named procumbenoside A, as well as eleven already-reported compounds. Their bioactive evaluation assays found that justicidin A (5), diphyllin (3), and tuberculatin (4) showed obvious cytotoxic effects. In addition, compounds **1** and **4** increased tumor-necrosis factor-α (TNF-α) production in RAW 264.7 cells treated with lipopolysaccharide (LPS) at low concentrations [11]. However, the selectivity of these compounds toward non-tumor cells was not described.

**Figure 4:**
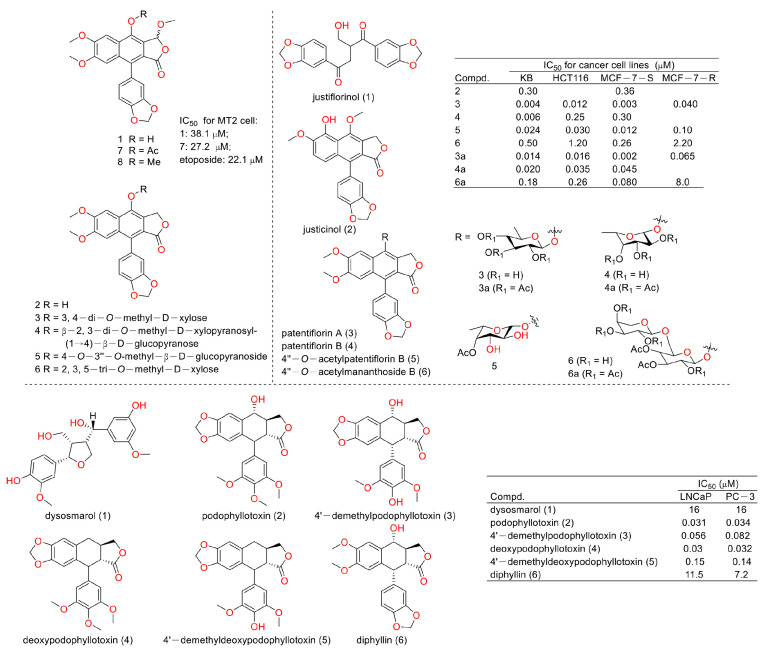
Structures and activities of diphyllin as well as its derivatives.

In 2003, Chimmani Ramesh et al. found that arylnaphthalide lignans from *Cleistanthus collinus* displayed cytotoxicity against the MT2 cell line (a kind of T lymphocytic leukemia). The IC_50_ values of cleistanone (1) and its acetyl derivative 7 (as showed in Figure 4) were 38.1 μM and 27.2 μM for MT2 cells, which was almost the same as the positive control etoposide (the IC_50_ value was 22.1 μM) [12]. In 2005, Sophie Susplugas et al. obtained one new norlignan (1) and five new lignans (2–6) from the leaves and stems of *Justicia patentiflora*. They found that lignans 2–6 displayed obvious cytotoxicity toward four cancer cell lines with IC_50_ values at nanomoles. They also led to G0/G1 phase blockades within cancer cells [13]. In 2007, Ren-Wang Jiang et al. reported lignans from *Dysosma wersipellis,* which showed anti-prostate cancer (LNCaP and PC-3 cells) activity with IC_50_ values ranging from 0.031 µM to 0.15 µM for compounds **2**–**5** [14].

**Figure 5:**
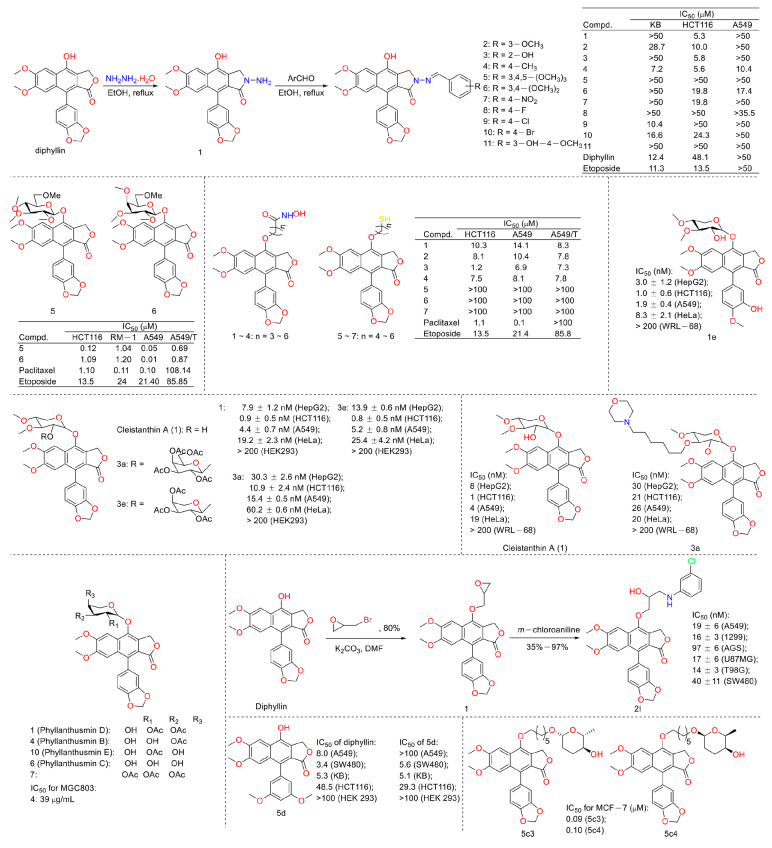
Structures and activities of diphyllin as well as its derivatives.

**Figure 6:**
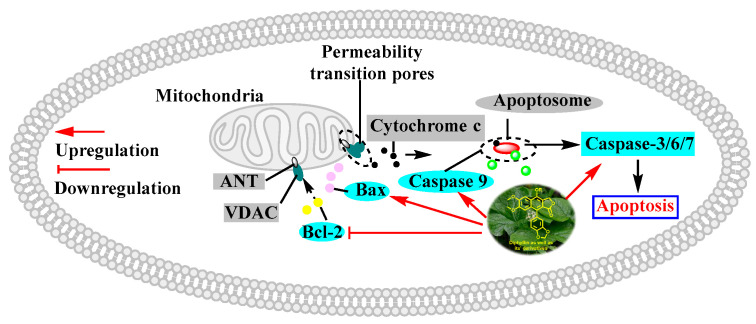
Diphyllin induced mitochondria-mediated apoptosis within cancer cells. Bcl-2: B-cell lymphoma-2. Bax: Bcl-2-associated X protein. VDAC: voltage-dependent anion channel. ANT: adenine nucleotide transporter.

**Figure 7:**
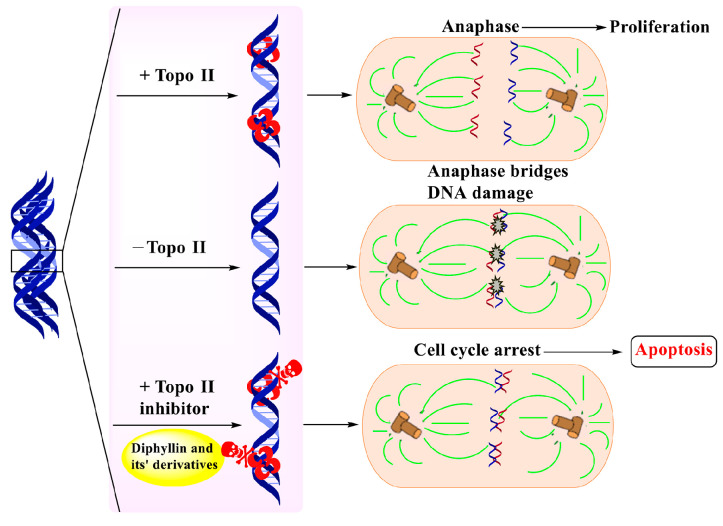
Diphyllin derivatives shown to have anti-tumor activity via topoisomerase II inhibition.

In 2010, Yu Zhao et al. performed a synthesis, anti-tumor evaluation, and mechanism investigation of benzoisoindolin hydrazones derived from diphyllin. This was the first study of the lactone derivatization of diphyllin. The results showed that derivatives 1, 3, and 4 (as shown in Figure 5) were more cytotoxic against HCT-116 (a kind of colon cancer cell line) compared with that of diphyllin. Significant mitochondrial-mediated apoptosis was induced by compound **4** within HCT-116 cells. It also decreased Bcl-2 (a protein that is necessary for cancer cell survival) and increased Bax expression (a cancer suppressor protein) (as displayed in Figure 6). This research demonstrated that the lactone was not a pharmacophore for the anti-tumor activity of diphyllin [15]. Two years later, these authors reported serials of novel diphyllin glycosides as anti-tumor agents again. In this study, they identified two novel lead compounds, **5** and **6**, which were more active than the positive control etoposide (an established Topo II inhibitor). Furthermore, they were active against the paclitaxel-resistant cell line, A549T (human lung cancer cells, which are resistant to Taxol). Mechanistic studies revealed that they acted on Topo II (as shown in Figure 7) and tubulin simultaneously to display their anti-tumor activity [16]. Then, in 2013, Yu Zhao et al. once again reported the anti-tumor activity of diphyllin hydroxamic acid and mercaptan hybridizations as Topo II and HADC1 dual inhibitors for the pursuit of effective anti-tumor agents, though the exact targets were far from verified. Their results showed that diphyllin hydroxamic acid hybridization exhibited obvious cytotoxicity against both sensitive and drug-resistant cancer cell lines, among which compound **3** possessed the best anti-colon cancer (HCT116 cells) potency with an IC_50_ value of 1.2 μM [17]. In the same year, Yu Zhao et al. reported novel arylnaphthalene lignans as anticancer agents. They screened out a lead 5d, which induced mitochondrial-mediated apoptosis within oral epidermoid cancer KB cells (IC_50_ value was 5.1 μM) with an increase in Bax and a decrease in Bcl-2 [18]. Two years later, Yu Zhao et al. also identified cleistanthin A derivatives as potent vacuolar H^+^-ATPase (V-ATPase, which was critical for managing cancer development, progression, and metastasis) inhibitors and neutralized the pH of lysosomes at submicromolar concentrations. Among these derivatives, compounds **3a** and **3e** showed the best activity (IC_50_ at ten micromole), though they were less active compared to cleistanthin A [19]. In the same year, they reported cleistanthin-A heterocyclic hybridization, which possessed antiproliferative effects on HepG2, HCT-116, A549, and HeLa (a human cervical carcinoma cell) at sub-micromolar concentrations, with selectivity toward normal hepatocyte WRL-68 at 200 nM. The lead compound **3a** also showed vacuolar H^+^-ATPase inhibitory activity [20]. This evidence implies that the inhibitors of V-ATPases may represent a feasible option as an anticancer strategy [21].

In 2016, Yapeng Lu et al. reported the mechanism of a previously reported vacuolar H^+^-ATPase inhibitor diphyllin glycoside derivative, ZT-25, which triggered apoptosis and protective autophagy via reactive oxygen species (ROS) generation within hepatoma carcinoma HepG2 cells. It induced a G1/G0 phase blockade, mitochondrial membrane potential (MMP) dissipation, and ATP depletion. The expression levels of Bcl-2 were downregulated, while those of Bax and cleaved caspase-3 were upregulated. In addition, autophagy was induced, as evidenced by an increase in the conversion of LC3 I to LC3 II, Beclin-1 expression, and autophagosome formation, along with a decrease in p-mTOR [22]. Additionally, in 2016, Yu Zhao’s group synthesized novel lignan glycosides as anticancer agents. They screened out a lead 1e, which exhibited strong anti-tumor potency with IC_50_ values ranging from 1.0 nM to 8.3 nM. It also displayed vacuolar H^+^-ATPase inhibitory activity at 60 nM (500 nM for diphyllin), as evidenced by the lysosome acidity assay [23]. In 2019, Zhao Yu’s group also reported the cytotoxicity of five natural diphyllin L-arabinopyranosides: Phyllanthusmin D (1), Phyllanthusmin B (4), Phyllanthusmin E, Phyllanthusmin C (6), and 7-*O*-[(2,3,4-tri-*O*-acetyl)-α-Larabinopyranosyl)] diphyllin (7) (structures are shown in Figure 5). Their bioassay results showed that compound **4** displayed the best cytotoxicity toward human gastric carcinoma MGC803 cells with an IC_50_ value of 39 μg/mL. Compounds **1** (67 μg/mL), **4** (37 μg/mL), and **7** (100 μg/mL) also suppressed MGC-803 cell invasion [24]. In 2021, Zhao Yu’s group designed and synthesized serials of diphyllin β-hydroxyl amino derivatives and evaluated their anti-tumor activities as well as V-ATPase inhibitory potency. They found that compound **2I** displayed the best anti-tumor activity, with IC_50_ values ranging from 14 nM to 97 nM for A549 (lung cancer cell line), NCI-H1299 (human non-small-cell lung cancer cell line), AGS (gastric adenocarcinoma cell line), U87MG (brain glioma cell line), T98G (brain glioma cell line), and SW480 (colon cancer cell line). They also found that these compounds demonstrated V-ATPase inhibitory activities, which matched their cytotoxicity well. In addition, 2I induced a decline in cytoplasmic pH in AGS cells in a concentration-dependent manner, indicating the promotion of cytosolic acidification [25]. In 2022, Zhao Yu’s group performed the synthesis and bio-evaluation of 2, 4, and 5-trideoxyhexopyranosides derivatives of diphyllin. They found that 5c3 and 5c4 (Figure 5) showed the best anti-breast cancer (MCF-7 cell line) potency with IC_50_ values of 0.09 μM and 0.10 μM, respectively. They also showed similar vacuolar H^+^-ATPase inhibitory activities to diphyllin [26].

**Figure 8:**
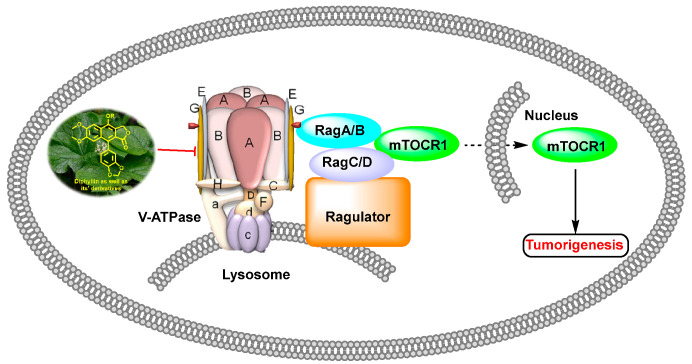
Diphyllin as well as its derivatives show anti-tumor activity via vacuolar H^+^-ATPase inhibition pathway. mTORC: mammalian target of rapamycin.

**Figure 9:**
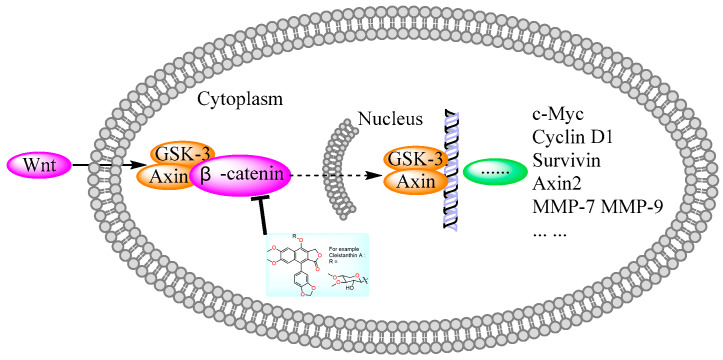
Diphyllin derivatives show anti-tumor activity via the Wnt/β-catenin pathway. Wnt: Wingless/Integrated; GSK: Glycogen Synthase Kinase; MMP: Matrix Metalloproteinase.

**Figure 10:**
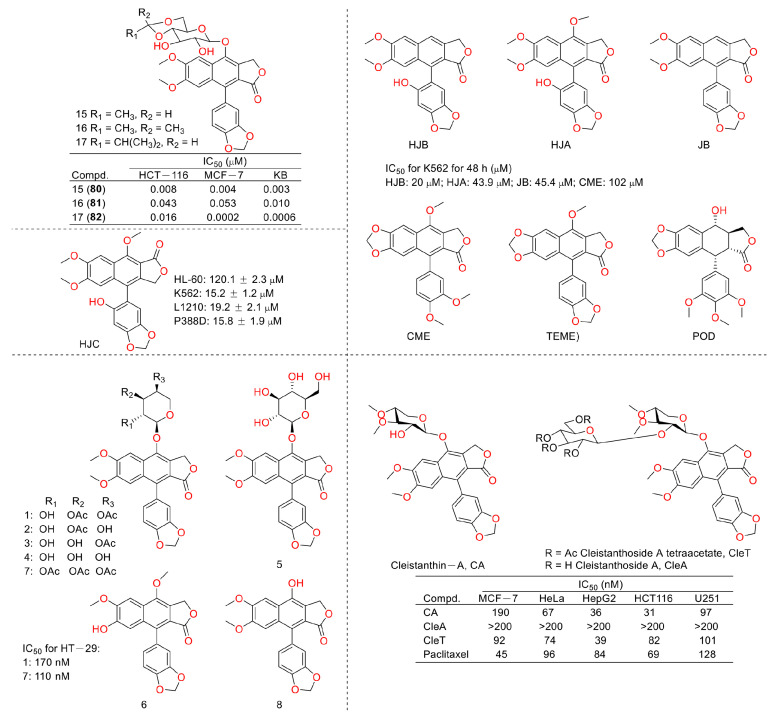
Structures and activities of diphyllin as well as its derivatives.

In 2011, Weidong Shen et al. demonstrated that diphyllin exhibited anti-gastric adenocarcinoma (SGC7901) activity via targeting vacuolar H^+^-ATPase (as displayed in Figure 8). Their mechanistic studies showed that diphyllin decreased phospho-LRP6 (instead of LRP6) and β-catenin in Wnt/β-catenin signaling and its two target genes (*c-Myc* and *cyclin D1*) (as vividly displayed in Figure 9) with the inhibition of vacuolar H^+^-ATPase [27]. In 2012, Dakuo Shi et al. performed the synthesis of novel glycosylated diphyllin derivatives as topoisomerase II-based anti-tumor agents. Based on their previously reported compound **11**, which exhibited anticancer activity, they synthesized twelve novel glycosylated diphyllin derivatives. Among these compounds, they found that compound **15** (structures are shown in Figure 10) showed the best anti-promyelocytic leukemia (HL-60 cells) activity with G0/G1 arrest and DNA fragmentation. Furthermore, compound **15** also displayed anticancer potency in muti-drug-resistant cancer cell lines (e.g., KB/VCR (oral epidermoid cancer cells, which are resistant to vincristine) and K562/A02 (chronic myeloid leukemia cells, which are resistant to doxorubicin)). The reversal index values were 29.3 and 19.4 for these two cell lines. They also drew the structure–activity relationship (SAR), highlighting that (1) sugar moiety is vital for anticancer potency; (2) the equatorial C-4′-OH on the sugar is preferred to the axial one; and (3) a proper cyclic lipophilic group at the C-4′ and C-6′ of sugar is beneficial [28]. In 2014, Jiaoyang Luo and colleagues first reported a novel arylnaphthalene lignan (HJC) (Figure 10) isolated from *Justicia procumbens*. It showed anti-leukemia activity (the IC_50_ values were 20.1 ± 2.3, 15.2 ± 1.2, 19.2 ± 2.1, and 15.8 ± 1.9 µM against HL-60, K562, L1210 (mouse leukemia cells), and P388D1 (mouse lymphoid tumor cells), respectively). They then performed a mechanism investigation and found that it induced K562 cell apoptosis via activating the caspase 3 cascade. It inhibited cell proliferation, reduced superoxide dismutase (SOD) activity, enhanced the ROS levels, and led to apoptosis. In the same year, these authors also reported the SAR of a series of arylnaphthalene lignans as anti-tumor agents. The IC_50_ values of HJB, HJA, JB, and CME against K562 for 48 h were 20, 43.9, 45.4, and 106.2 µM, respectively. The anti-tumor activity orders were as follows: HJB > HJA > JB > CME > TEME. HJB, HJA, and JB (Figure 10) all reduced the SOD activity and induced caspase 3-dependent apoptosis in leukemia K562 cells. Compared with these results, they found that hydroxyl substitution at C-1 and C-6′ of arylnaphthalene lignans was favored, while a methoxyl at C-1 was significantly unfavored [29]. In 2015, Yulin Ren et al. reported some novel (1 and 2) (Figure 10) and already-reported arylnaphthalene lignans (3-8) with potent anticancer activities both in vivo and in vitro. Among them, compounds **1** and **7** showed the most potent anti-HT-29 human colon cancer abilities, with IC_50_ values of 170 and 110 nM, respectively. The mechanistic studies revealed that the anti-tumor activities of these two leads were related to caspase 3-dependent apoptosis without affecting Topo II [30]. In 2014, Zhitao Zhang et al. reported diphyllin glycosides as vacuolar H^+^-ATPase-based anticancer agents. Cleistanthin-A and Cleistanthoside A tetraacetate (Figure 10) showed antiproliferative potency against MCF-7, HeLa, HepG2, HCT-116, and U251 cells with IC_50_ values at nanomolar concentrations. They also displayed vacuolar H^+^-ATPase inhibition activity within HepG2 cells [31].

**Figure 11:**
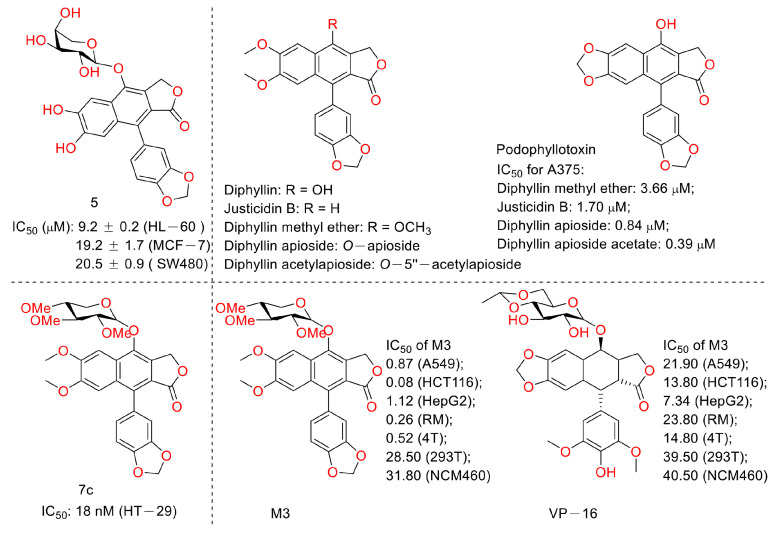
Structures and activities of diphyllin as well as its derivatives.

In 2015, Shan Yu et al. and co-workers reported cytotoxic lignan glycosides from *Phyllanthus glaucus*. They found that the reported lignan glycoside 5 (Figure 11) displayed the best activity, with IC_50_ values of 9.2 ± 0.2, 19.2 ± 1.7, and 20.5 ± 0.9 μM for HL-60, MCF-7, and SW480 cells [32]. In 2017, Aljawharah Al-Qathama et al. disclosed that the derivatives of Justicidin B (Figure 11) displayed anti-melanoma (A375 cells) activity via Bax/Bcl-2 ratio augmentation and caspase-3/7 activation [33]. In 2017, Sheng Pan and co-workers first reported that Cleistanthin A (Figure 5) blocked the invasion and metastasis of human melanoma cells (A537) by inhibiting the expression of matrix metallopeptidase-2 and -9 (MMP-2, -9) in dose-dependent (0.03, 0.1, and 0.3 µM concentrations, at which cell viability was not affected) and time-dependent manners. It also inhibited the activity of V-ATPases, increased the acidity of the cytoplasm, and increased the alkalinity of the lysosome in A375 cells [34]. In 2018, Haijiao Chen et al. reported the V-ATPase-based anti-esophageal cancer (TE-1 and ECA-109 cell lines) activity of diphyllin. It led to S-phase arrest within these two tested cancer cell lines. The mechanistic studies found that diphyllin (concentrations of 0.3125-30 μM) decreased the mRNA expression of the mammalian targets of rapamycin complex 1 (mTORC1), hypoxia-inducible factor-1α (HIF-1α), and vascular endothelial growth factor (VEGF). It (concentrations of 0.3125-30 μM) also reduced the formation of new blood vessels to reduce the blood metastasis via the mTORC1/HIF-1α-/VEGF pathway [35]. In 2019, Weidong Shen et al. reported a natural lignan xyloside M3 (Figure 11), which displayed anti-HCT116 activity (the IC_50_ value was 0.08 μM). It induced caspase 3-dependent apoptosis. The mechanistic studies revealed that the anticancer activity was associated with the promotion of microtubule depolymerization, similar to that of taxol [36]. In 2018, John L. Woodard et al. performed a synthesis and antiproliferation evaluation of the phyllanthusmin class of arylnaphthalene lignan lactones. They found that the lead compound, **7c**, possessed an IC_50_ value of 18 nM against colorectal adenocarcinoma HT-29 cells (the IC_50_ values of several others ranged from 50 nM to 200 nM). The mechanistic studies found that their antiproliferative potency was not related to Topo II inhibitory activity [37].

**Figure 12:**
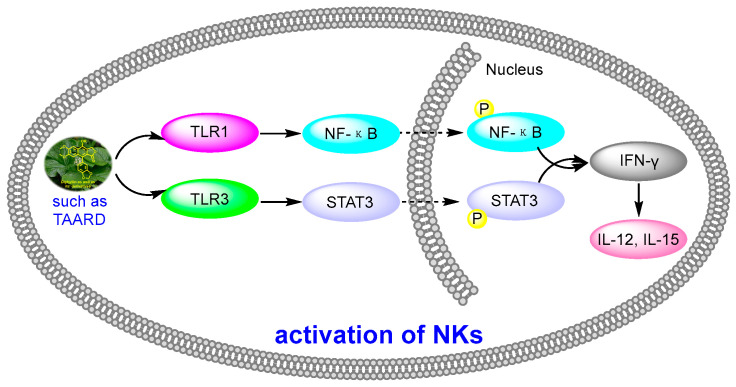
Activation of NKs by diphyllin derivatives such as TAARD via TLR1/NF-κB and TLR3/STAT3 pathways.

Human natural killer cells (NKs) are capable of destroying tumor cells directly. However, until 2018, no scientists had reported the role of natural products in activating NKs. In 2018, Long Yi et al. reported the activation of NKs by a synthetic disaccharide derivative of diphyllin (named TAARD), which stimulated interferon (IFN)-γ secretion in NKs and increased IL-12 or IL-15 in IFN-γ production via TLR1/NF-κB and TLR3/STAT3 pathways (elevating phosphorylation levels, as shown in Figure 12) as a result of binding on the IFNG promote. These results implied that TAARD is promising for cancer prevention or treatment [38]. In 2019, Siyuan Liu et al. reported that a natural diphyllin glycoside, Cleistanthin A (CleA (Figure 5), at concentrations of 0.1, 0.3, and 1 μM, suppressed the invasion of MDA-MB-231 cancer cells in a concentration-dependent manner without significant cell death via the β-catenin pathway. The expression and nuclear translocation of β-catenin were also decreased via CleA (Figure 5). Further mechanism investigations showed that the expression and activation of MMP-2/9, downstream targets of the β-catenin pathway, were decreased. Subsequently, the transcription of the *cyclin D1* and *c-Myc* genes, two well-known downstream genes of the β-catenin pathway, was inhibited. Further studies have highlighted that this β-catenin pathway inhibition was related to the promotion of β-catenin degradation via the inhibition of GSK3β phosphorylation without affecting the β-catenin mRNA levels. This study, along with previous reports, demonstrated that it not only acted as a regulator of the acidic tumor microenvironment but also as an inhibitor of the β-catenin pathway (as shown in Figure 9) [39].

**Figure 13:**
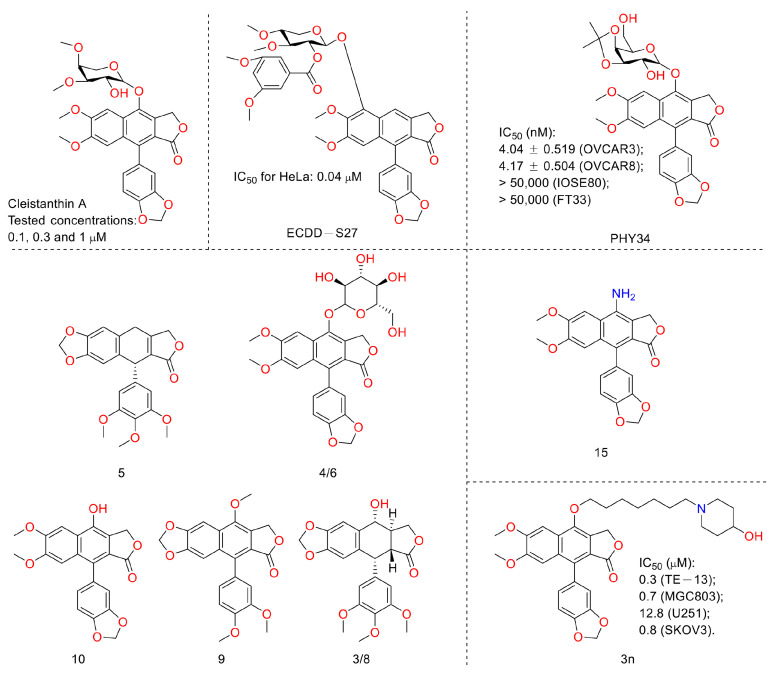
Structures and activities of diphyllin as well as its derivatives.

In 2019, Jiraporn Paha et al. reported that the diphyllin glycoside derivative (named ECDD-S27) (Figure 13) induced autophagy by targeting vacuolar ATPase, increased levels of p62 and LC3-II, and ultimately led to cancer cell survival inhibition (IC_50_ ranged from 0.016 µM to 0.080 µM). ECDD-S27 increased the number of autophagic vacuoles by restraining autophagic flux and inhibiting V-ATPase activity and consequently decreased colorectal adenocarcinoma HT-29 cell viability at concentrations ranging from 0.016 μM to 0.4 μM. It is also nontoxic to human kidney normal HK-2 cells with an IC50 value higher than 50 μM [40]. In 2021, Huixia Feng et al. demonstrated the anti-tumor activity of diphyllin. It possessed IC_50_ values of 6.46 ± 1.79 μM and 30.73 ± 0.56 μM on A549 and HT-29 cells, respectively. It also showed good COX-2 inhibitory activity, with an IC_50_ value of 1.29 ± 0.14 μM. Furthermore, other bioactive components (diphyllin derivatives for example) (Figure 13) from *Podophyllum sinense* were also identified by researchers using multi-target ultrafiltration [41]. One year later, Yang Li et al. designed and synthesized novel nitrogen-containing derivatives of diphyllin as anti-tumor agents. They screened out a lead 15 whose IC_50_ values toward pancreatic cancer CFPAC-1 cells were 3 nM (69-fold more potent than that of diphyllin). It showed selectivity toward normal hepatocyte L02 cells with a selective index of 162. In addition, it possessed better drug-like properties (e.g., improved aqueous solubility and metabolic stability in liver microsomes). The mechanistic studies revealed that the lead induced a G0/G1 phase blockade and decreases in CDK4 and cyclin D1 in a concentration-dependent manner. It restricted the later stage of autophagy in CFPAC-1 cells, too. Furthermore, it (10 mg/kg) displayed obvious anti-PANC02 (mouse pancreatic cancer) in vivo without obvious toxicity to the mice [42].

Last year, Amrita Salvi et al. announced a previously reported [43] synthetic compound, PHY34, which displayed anti-high-grade serous ovarian cancer (HGSOC) activity, both in vivo and in vitro. It induced apoptosis in ovarian cancer cells via late-stage autophagy inhibition. They identified a target cellular apoptosis susceptibility (CAS) protein, also known as CSE1L, via mass spectrometry-based chemoproteomics. Their further studies highlighted that it targeted the ATP6V0A2 subunit to induce autophagy inhibition and interacted with CAS to alter the nuclear localization of proteins [44]. In 2022, Weidong Shen et al. performed the work of synthesis, cytotoxicity, anti-migration, and anti-invasion activity toward the MGC-803 (gastric carcinoma cells) of diphyllin heterocyclic derivatives. They screened a lead compound, **3n**, that showed anti-migration and invasion abilities and inhibited the V-ATPase on MGC-803 cells at 0.75 μM. The IC_50_ values were submicromole for MGC-803, U251 (glioma cells), and SKOV3 (ovary carcinoma cells) [45]. In 2022, Sagar Puli et al. reported the antiproliferative and anti-migratory activities of diphyllin (**1**) on human colorectal cancer cells. The IC_50_ values were 2.9 ± 0.38, 1.3 ± 0.28, and 3.9 ± 0.65 µg/mL against HT-29, SW-480, and HCT-15 (colorectal adenocarcinoma cells), respectively. The mechanistic studies revealed that diphyllin induced apoptosis within cancer cell lines [46].

**Figure 14:**
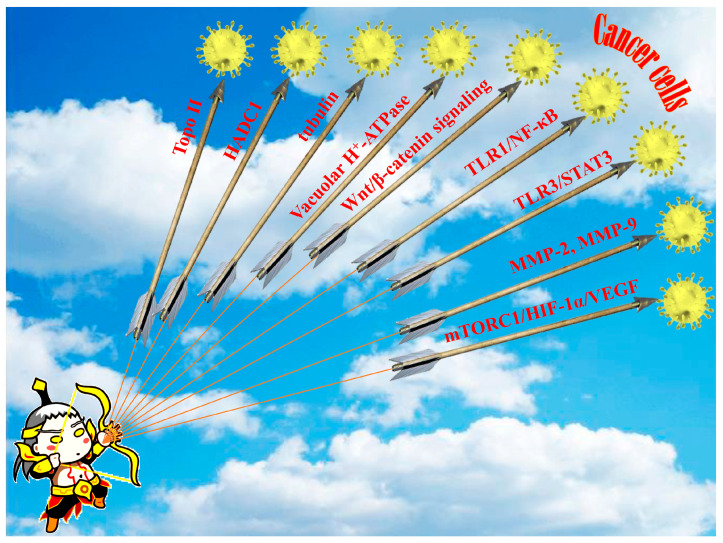
The main regulatory pathways or targets of the anti-tumor activity of diphyllin and its derivatives. The inspiration for the creation of this figure is a combination of Chinese mythology, namely, Houyi shooting the suns.

In total, the biological test assays and anti-tumor mechanisms of diphyllin as well as its derivatives are summarized in Table 1. In a word, diphyllin and its derivatives showed wide anti-tumor activity via multiple mechanisms, as shown in Figure 14, though most of the studies did not report selectivity to the non-cancer cell lines of these compounds.

## 3. Anti-Virus Activity

**Figure 15:**
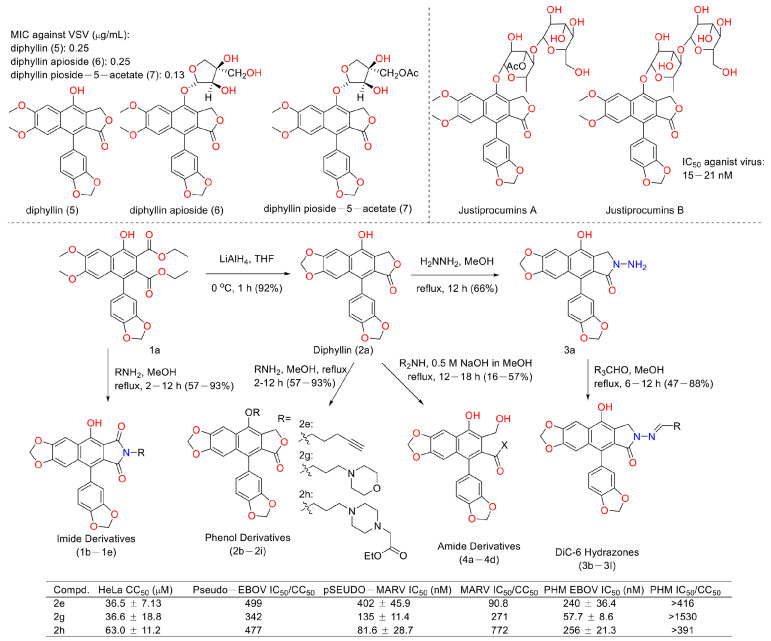
Structures and activities of diphyllin as well as its derivatives.

Viruses such as SARS-CoV-2 brought disaster to the world for people of all ages [48]. Therefore, the discovery of anti-virus drugs is meaningful for human health. In 1996, Jun, Asano et al. found that diphyllin (5), diphyllin apioside (6), and diphyllin pioside-5-acetate (7) (Figure 15) showed anti-virus activities, with MIC values lower than 0.25 μg/mL for the vesicular stomatitis virus, though the mechanism of action was unknown. More importantly, they showed selectivity toward RL-33 (a rabbit lung cell line), with MTC values larger than 31 μg/mL. They also displayed the same effects toward the Sindbis virus and the murine cytomegalovirus [49]. In 2012, Hui-Wen Chen et al. announced that diphyllin showed anti-influenza virus activities *via* targeting vacuolar H^+^-ATPase (an important regulating factor in influenza virus replication) in a dose-dependent manner. The combination of host-targeting diphyllin with pathogen-targeting therapeutics (oseltamivir and amantadine) was synergetic. The 50% cytotoxic concentration (CC_50_) values of diphyllin toward MDCK cells and in A549 cells were 3.48 ± 0.17 μM and 24.01 ± 0.45 μM (72 h), respectively. It (0.078, 0.312, and 1.25 μM) also inhibited endosomal acidification in both cell lines. Pretreatment with 2 μM diphyllin decreased the cellular susceptibility to influenza virus. The replication of the H6N1 avian influenza virus and dengue virus serotype 2 was also inhibited by diphyllin (2 μM). It (0.125-1 μM) also inhibited the proliferation of seasonal H1N1, the 2009 pandemic H1N1, two reference strains of H3N2 and type B influenza virus, and a plaque-purified DENV2 strain. This study shows that diphyllin (**1**) possesses a wide spectrum of antiviral activity [50]. Hence, scientists are curious about the anti-SARS and anti-COVID-19 (two broad-spread contagion that brought vast damage to human beings in 2003 and 2020) potency of diphyllin. In 2017, Che-Ming Jack Hu et al. reported the anti-feline coronavirus activity of diphyllin (the CC_50_ was 5.99 ± 0.68 μM) as well as its nanoformulation (the CC_50_ was 77.26 ± 13.14 μM). The effects were the most prominent when the fcwf-4 cells were exposed to diphyllin prior to FIPV exposure [51]. One year later, they also reported the anti-influenza virus infection activities of diphyllin and bafilomycin nanoparticles. Sustained drug release kinetics beyond 72 h and facilitated intracellular drug delivery to two different influenza virus-permissive cell lines were observed in the nanoparticle-treated groups (3- and 5-fold more effective than diphyllin- and bafilomycin-treated groups). It also reduced body weight loss and viral titer in the lungs of mice in a mouse model of the sublethal influenza challenge [52]. In 2017, Hongjie Zhang et al. reported that diphyllin glycosides from *Justicia gendarussa* displayed anti-HIV virus activity. These authors used a bioassay-guided method to identify two anti-HIV compounds, justiprocumins A and B, from the fractionation of the methanol extract of the stems and barks of *Justicia gendarussa*. Justiprocumin B displayed a broad spectrum of anti-virus activity, with IC_50_ values of 15–21 nM (AZT, IC_50_ 77–95 nM). It also showed anti-virus activity toward HIV-1_1617-1_ (nucleoside reverse transcriptase inhibitor-resistant isolate, which is resistant to AZT and HIV-1_N119_ (non-nucleoside reverse transcriptase inhibitor-resistant isolate, which is resistant to nevaripine)) [53]. In 2018, Aaron Lindstrom et al. synthesized four series of diphyllin derivatives and evaluated their inhibitory potency on replication-competent Ebola viral entry into primary macrophages. Additionally, they found that the three compounds (**2e**, **2g**, and **2h**) displayed good activity with high selectivity (selectivity index is >100) [54].

**Figure 16:**
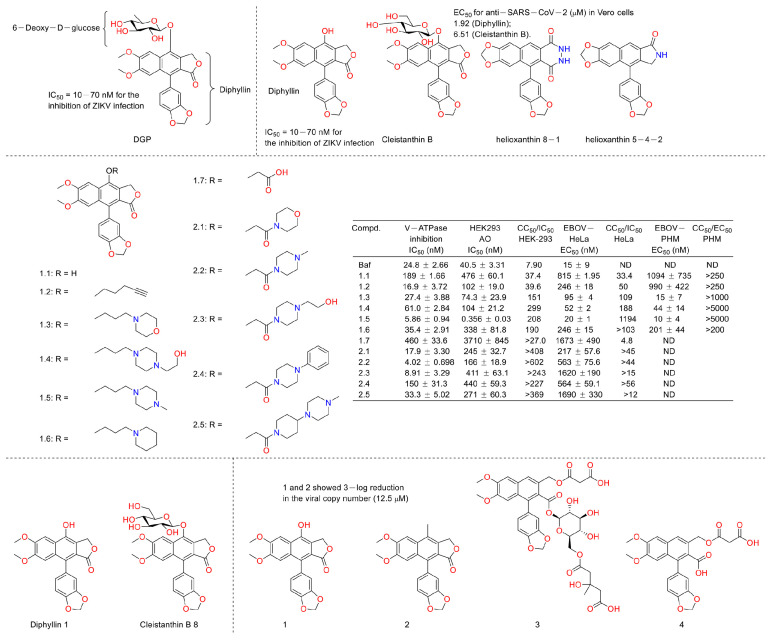
Structures and activities of diphyllin as well as its derivatives.

Numerous studies have suggested that ZIKV infection was obviously affected by the inhibition of endosomal acidification [55,56,57]. Inspired by the fact that diphyllin exhibited vacuolar H^+^-ATPase inhibition, Alicia Martinez-Lopez et al. evaluated the anti-ZIKV infection activity of 6-deoxyglucose-diphyllin (DGP) (Figure 16). They found that diphyllin (**1**) exhibited anti-ZIKV activity, both in vitro and in vivo (1 mg/kg), by preventing the acidification of endosomal/lysosomal compartments in different monkey and human cell lines without triggering cellular toxicity and then by inhibiting ZIKV fusion with cellular membranes and infections (IC_50_ = 10–70 nM for the inhibition of ZIKV infection) in 2019. It also displayed broad spectrum antiviral activity against other flaviviruses [58]. Last year, Michal Stefanik et al. designed assays to investigate the anti-SARS-CoV-2 activity of diphyllin (1) and diphyllinoside cleistanthin B and two structurally related compounds, helioxanthin 8-1 and helioxanthin 5-4-2. Their results showed that only diphyllin (1) and diphyllinoside cleistanthin B displayed anti-SARS-CoV-2 activity with EC_50_ values of 1.92 and 6.51 µM in Vero cells without cytotoxicity at concentrations of up to 100 μM [59]. In 2022, Caroline B. Plescia et al. performed a synthesis and bio-evaluation of phenol-substituted diphyllin derivatives. Their results showed that these derivatives inhibited viral entry that was dependent upon structural variations at nanomolar concentrations in cells challenged with the Ebola virus. The mechanism research revealed that they modulated the endosomal pH. These results were consistent with the fact that diphyllin is a selective pH-dependent viral entry block in late endosomes [60]. In 2022, Michal Štefánik et al. reported that diphyllin and diphyllinosides (Cleistanthin B 8) displayed broad-spectrum antiviral activities against multiple enveloped RNA and DNA viruses by blocking the replication of these viruses. Diphyllin showed antiviral activity against tick-borne encephalitis virus, West Nile virus, Zika virus, Rift Valley fever virus, rabies virus, and herpes-simplex virus type 1 at sub-micromolar or low-micromolar concentrations. It was not cytotoxic for Vero and BHK-21 cells up to 100 µM [4]. This year, Gergö Tóth et al. evaluated the antiviral and cytotoxic potency of two known arylnaphthalene lignans (diphyllin and justicidin B (2)) and two novel arylnaphthalene lignans (linadiacin A (3) and linadiacin B (4)) in SARS-CoV-2-infected cells and various cancer cell lines in vitro. They found that both of them showed anti-SARS-CoV-2 activities with a 3-log reduction in the viral copy number at a 12.5 μM concentration. Some of them displayed obvious cytotoxicity against cancer cell lines at several micromoles (2.4 μM and 2.6 μM against A2058 for 3 and 4, respectively, and 2.55 μM against MM6 for 2) [61]. The biological test assays and anti-virus mechanisms of diphyllin as well as its derivatives are summarized in Table 2.

## 4. Other Biological Activities

**Figure 17:**
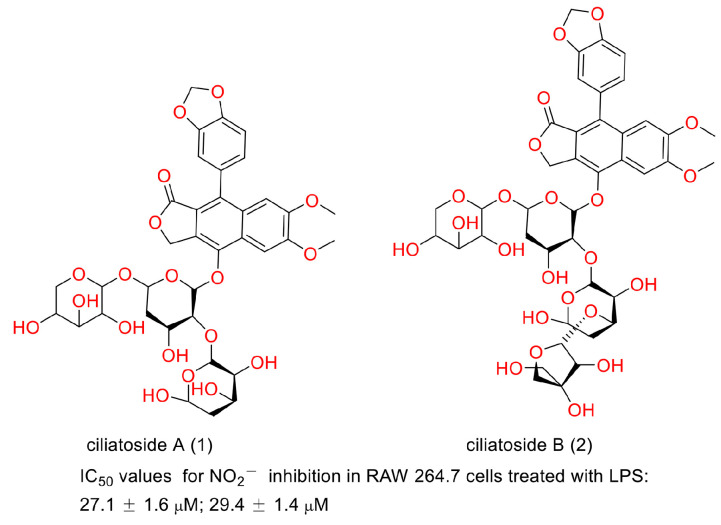
Structures and activities of ciliatoside A and ciliatoside B.

In 2020, Suman Thamburaj et al. reported the antibacterial and antibiofilm activities of diphyllin against fish pathogens. They found that diphyllin disrupted *Salmonella typhi* biofilms by producing ROS at concentrations of 25, 50, 75, and 100 μg/mL. It caused *S. typhi* cell membrane damage and intracellular DNA degradation. It showed promise in combating the deleterious effects on food products brought about by bacteria and biofilms [62]. Biofilm is conducive to drug-resistance bacteria generation, persistent regrowth, and chronic infections [63,64]. Therefore, diphyllin or its derivatives may show promise in these situations. In 2000, Shiow-Hwa Day and co-workers announced that two new lignan glycosides (ciliatoside A (1) and ciliatoside B (2)) (Figure 17) displayed obvious anti-inflammation activities. They possessed IC_50_ values of 27.1 ± 1.6 and 29.4 ± 1.4 µM for NO_2_^−^ inhibition in RAW 264.7 cells treated with LPS, respectively [65]. In 2020, Ya-Nan Duan et al. reported that diphyllin alleviated high-fat-diet-induced obesity in mice through brown and beige adipocytes. It promoted C3H10-T1/2 cell and primary brown/beige preadipocyte differentiation and thermogenesis, leading to increased energy consumption. The treatment of 100 mg/kg diphyllin ameliorated the oral glucose tolerance and insulin sensitivity and decreased the body weight and fat content ratio. It also augmented adaptive thermogenesis in HFD-fed mice under cold stimulation and whole-body energy expenditure. This study provided new clues for the discovery of anti-obesity molecules from natural products such as diphyllin [66]. In 2018, Henrik Löfvall et al. reported that vacuolar-type H^+^-ATPase (V-ATPase) inhibitor diphyllin showed bone resorption inhibitory potency. Diphyllin inhibited osteochondral CTX-I release, decreased the Ca^2+^ concentration, increased TRAP activity, and increased cell viability at a concentration of 30 nM. It showed promise for the treatment of rheumatic diseases [67]. Factually, many bioactivities of diphyllin and its derivatives, as have been described above, are related to this characteristic. The biological test assays and mechanisms of other biological activities of diphyllin as well as its derivatives are concluded in Table 3.

## 5. Conclusions and Perspective

**Figure 18:**
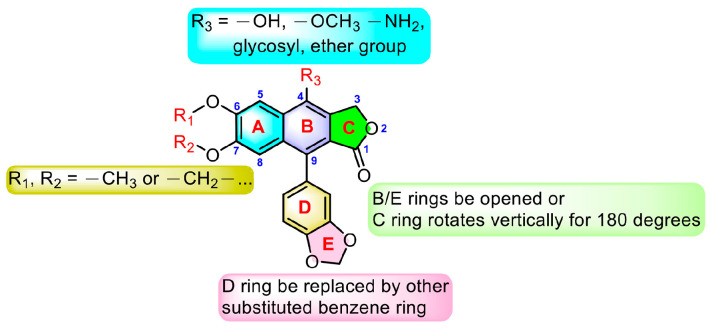
Modification strategies of diphyllin.

From the above-mentioned research results, we can conclude the modification strategies of diphyllin (Figure 18). The most common strategy is modification at C-4. This strategy includes glycosylation, hydroxylation, methoxylation, and amination at C-4. Additionally, glycosylation will largely enhance the bioactivity of diphyllin, probably due to the elevated water solubility or membrane permeability. Furthermore, the absolute configuration of C-4′-OH on the sugar is an influential factor for bioactivity. A proper cyclic lipophilic group at the C-4′ and C-6′ of sugar is also meaningful. Modification at C-6 and C-7 (such as methoxylation and dioxanation) is also frequently seen in some reports. Some research groups also replaced the D ring with another substituted benzene ring. The opening of the B ring and the vertical rotation of the C ring would occasionally bring advantages.

Together, many studies demonstrated that diphyllin, along with its derivatives, showed several cellular functions, including vacuolar H^+^-ATPase activity inhibition [27,35], endosomal acidification inhibition [68], and topoisomerase IIα inhibition [16,28]. As is known to us all, V-ATPases are distributed in the vesicles, lysosomes, endoplasmic reticulum, and other organelles of some organs, tissues, or cells, the function of which is to pump protons from the intracellular to the extracellular area, or from the inner membrane to the intermembrane layer. It is precisely the presence of V-ATPases through which tumor cells can transport a large amount of H^+^ produced in the metabolism to the outside of the cell and maintain the neutral cytoplasm and extracellular acidic environment so as to avoid causing their own acidosis. These H^+^ discharged outside the tumor cells will enter the normal cell tissues with the concentration gradient and accumulate in large quantities, activating the enzyme cascade reaction leading to cell necrosis or apoptosis, which is more conducive to the spread and metastasis of the tumor. In addition, studies have shown that the acidic environment of tumor microenvironments can induce increased lysosome secretion and activation and activate proteolytic enzymes to promote the degradation and remodeling of the extracellular matrix (ECM), which contributes to tumor invasion and metastasis [69]. In addition, this enzyme is also related to membrane traffic, protein degradation, autophagy, and the coupled transport of small molecules. These are important for pathogen entry, cancer cell invasiveness, and neurodegenerative diseases, as well as other diseases such as osteopetrosis [70,71]. It is not hard to find that many of the above-mentioned bioactivities (e.g., anti-tumor (HepG2, A549, 1299, AGS, U87MG, T98G, SW480, SGC7901, MCF-7, HeLa, HepG2, HCT-116, U251, HT-29, and MGC-803), anti-SARS-CoV2, and anti-rheumatic diseases) of diphyllin and its derivatives are closely related to V-ATPases inhibition. These results imply that diphyllin and its derivatives may display anti-neurodegenerative activities. Selectivity is an important evaluation factor for an anti-tumor agent. Though many studies have reported the anti-tumor activities of diphyllin and its analogues, most of them did not uncover selectivity toward the non-cancer cells of these compounds except for the studies by Yu Zhao et al. [20], Jiraporn Paha et al., [40] and Yang Li et al. [42].

As is well known, glycosylation-endowed compounds have good water solubility. Additionally, glycosylation products can easily pass through the cytomembrane [72]. Interestingly, diphyllin–carbohydrate hybrids have been widely studied, and the results have shown that some of these compounds are more active than diphyllin. This is instructive for our drug design and development, especially for the pursuit of elevated water solubility and cytomembrane transmission. It also cannot be ignored that some of the action targets were not revealed. This is a direction that is worth our attention and effort. The identification of action targets is a difficult problem to solve. Fortunately, many advanced methods and technologies have been put forward by scientists to solve this difficult problem. Chemical proteomics technology and techniques, such as photoaffinity chromatography and drug affinity-responsive target stability, are resourceful examples for target fishing [73,74]. We are confident that, in the future, many more mechanisms of action for diphyllin and its derivatives will be revealed and reported by research groups.

## Figures and Tables

**Table 1:** Biological test assays and anti-tumor mechanisms of diphyllin as well as its derivatives.

Compound Number in Original References	Biological Tests and Assays	Anti-Tumor Mechanisms	References
1, 5, and 7	MTT	unreported	[9]
diphyllin (3)	MTT	unreported	[10]
justicidin A (5), diphyllin (3), and tuberculatin (4)	MTT, ELISA kit	tumor necrosis factor-α augment	[11]
cleistanone (1), 7	MTT	unreported	[12]
2-6	MTT, flow cytometry, P388 leukemia model	G0/G1 phase blockade	[13]
2-5	MTS	unreported	[14]
1, 3, and 4	MTT, PI and EB staining, Western blot	mitochondrial-mediated apoptosis induction, Bcl-2 decrease, Bax increase	[15]
5 and 6	MTT, kDNA decatenation assay, tubulin polymerization assay	Topo II and tubulin inhibition	[16]
3	MTT, Western blot	Topo II and HADC1 inhibition	[17]
5d	MTT, Western blot	mitochondrial-mediated apoptosis induction, Bcl-2 decrease, Bax increase	[18]
3a and 3e	MTT, colorimetric assay	vacuolar H^+^-ATPase inhibition	[19]
3a	MTT, colorimetric assay	vacuolar H^+^-ATPase inhibition	[20]
ZT-25	MTT, Western blot	G1/G0 phase blockade, mitochondrial membrane potential dissipation, ATP depletion, Bcl-2 downregulation, Bax and cleaved caspase-3 upregulation. Autophagy induction, LC3 I to LC3 II, Beclin-1 increase, and p-mTOR decrease	[22]
1e	MTT, lysosome acidity assay	vacuolar H^+^-ATPase inhibition	[23]
1, 4, and 7	MTT, transwell invasion assay	unreported	[24]
2I	MTT, vacuolar H^+^-ATPase activity assay kit	vacuolar H^+^-ATPase inhibition	[25]
diphyllin, 5c3, and 5c4	MTT, vacuolar H^+^-ATPase activity assay kit, molecular docking	vacuolar H^+^-ATPase inhibition	
diphyllin	MTT, vacuolar H^+^-ATPase activity assay kit, Western blot, real-time PCR	Wnt/β-catenin signaling inhibition (phospho-LRP6 and β-catenin expression inhibition, *c-Myc* and *cyclin D1* gene downregulation)	[27]
15	Sulforhodamine B (SRB), MTT, DNA fragmentation electrophoresis, flow cytometry	topoisomerase II inhibition, G0/G phase arrest	[28]
arylnaphthalene lignan (HJC)	MTT, Western blot, real-time PCR	activation of caspase 3 cascade, SOD inhibition, ROS elevation	[47]
HJB, HJA, JB, and CME	MTT, SOD activity assay kit, flow cytometry, Western blot	SOD inhibition, ROS elevation, caspase-dependent intrinsic and/or extrinsic apoptosis pathways	[29]
1 and 7	MTT, Western blot, Topoisomerase II assay, hollow fiber assay	caspase 3-dependent apoptosis	[30]
Cleistanthin-A and Cleistanthoside A tetraacetate	MTT, vacuolar H^+^-ATPase activity assay kit	vacuolar H^+^-ATPase inhibition	[31]
5	MTT	unreported	[32]
Justicidin B	MTT, Western blot	Bax/Bcl-2 ratio augment and caspase-3/7 activation	[33]
Cleistanthin A	MTT, Western blot, vacuolar H^+^-ATPase activity assay kit	MMP-2 and MMP-9 inhibition, vacuolar H^+^-ATPase inhibition	[34]
diphyllin	MTT, real-time PCR	decreased the mRNA expressions of mTORC1, HIF-1α, and VEGF	[35]
7c	MTT	unreported	[37]
M3	MTT, Western blot, CytoDYNAMIX Screen 03 Tubulin Polymerization assay	caspase 3-dependent apoptosis, promotion of microtubule depolymerization	[36]
TAARD	MTT, Western blot	TLR1/NF-κB and TLR3/STAT3 pathway inhibition	[38]
Cleistanthin A	MTT, Western blot, real-time PCR	β-catenin pathway inhibition	[39]
ECDD-S27	MTT, immunoblot analysis	restrain of autophagic flux and inhibition of vacuolar H^+^-ATPase activity	[40]
diphyllin derivatives	MTT, UF-LC/MS screening assay, molecular docking	COX-2 inhibition	[41]
15	MTT, Western blot, flow cytometry, mouse pancreatic cancer model	G0/G1 phase blockade, CDK4, and cyclin D1 decrease	[42]
PHY34	MTT, mass spectrometry-based chemoproteomics	targeting ATP6V0A2 subunit to induce autophagy inhibition and interacting with CAS to alter the nuclear localization of proteins	[44]
3	MTT, transwell invasion and scratch wound assay, vacuolar H^+^-ATPase activity assay kit	vacuolar H^+^-ATPase inhibition	[45]
diphyllin	MTT, AO/EB dual staining assay, flow cytometry, wound-healing assay	apoptosis induction	[46]

**Table 2:** Biological test assays and anti-virus mechanisms of diphyllin as well as its derivatives.

Compound Number in Original References	Biological Tests and Assays	Anti-Virus Mechanisms	References
diphyllin (5), diphyllin apioside (6, and diphyllin pioside-5-acetate (7)	MIC test	unreported	[49]
diphyllin	MTT, acridine orange labeling assay, fluorescence microscopy assay, RT-PCR, Western blot, TCID_50_ assay, hemagglutination test, plaque assay, CPE inhibition assay	vacuolar H^+^-ATPase inhibition	[50]
diphyllin	MTT, ADE model of FIPV infection, blood chemistry analysis	vacuolar H^+^-ATPase inhibition	[51]
diphyllin	transmission electron microscopy and dynamic light scattering assay, cell cytopathic effect inhibition assay, mouse model of the sublethal influenza challenge	vacuolar H^+^-ATPase inhibition	[52]
justiprocumins A and B	luciferase gene reporter assay	unreported	[53]
2e, 2g, 2h	pseudovirus assays, MTT, vacuolar H^+^-ATPase inhibition	vacuolar H^+^-ATPase inhibition	[54]
6-deoxyglucose-diphyllin (DGP)	acridine orange staining assay, MTT, qRT-PCR	acidification of endosomal/lysosomal compartments prevention	[58]
diphyllin, diphyllinoside cleistanthin B, helioxanthin 8-1, and helioxanthin 5-4-2	viral titer reduction assay, MTT, plaque assays, immunofluorescence staining	unreported	[59]
diphyllin, diphyllinosides (Cleistanthin B 8)	acridine orange staining assay, GFP-EBOV virus infection assay, GFP-VLP uptake and trafficking assay, immunofluorescence staining, confocal imaging	vacuolar H^+^-ATPase inhibition	[60]
diphyllin, justicidin B (2), linadiacin A (3), linadiacin B (4)	MTT, TCID_50_ value determination	unreported	[61]

**Table 3:** Biological test assays and mechanisms of other biological activities of diphyllin as well as its derivatives.

Compound Number in Original References	Activity	Biological Tests and Assays	Mechanisms	References
diphyllin	antibacterial and antibiofilm	MIC, MBC, biofilm disruption assay, ROS and PI staining	membrane damage and intracellular DNA degradation, ROS generation	[62]
ciliatoside A and ciliatoside B	anti-inflammation	LPS-treated RAW 264.7 cell model	unreported	[65]
diphyllin	anti-obesity	SRB	differentiation and thermogenesis induction	[66]
diphyllin	anti-rheumatic diseases	colorimetric calcium assay, TRAP activity measurement, von Kossa staining	vacuolar-H^+^-ATPase inhibition, osteochondral CTX-I release inhibition, Ca^2+^ concentration and TRAP activity decrease, and cell viability increase	[67]

## Data Availability

No data were used for the research described in the article.
